# Editorial: Dietary protein for human health

**DOI:** 10.3389/fnut.2024.1542080

**Published:** 2025-01-15

**Authors:** Paul J. Moughan, Wouter H. Hendriks, Suzanne M. Hodgkinson, Sylvia M. S. Chungchunlam, Wen Xin Janice Lim, Marco Mensink, Natascha Stroebinger, Nikkie van der Wielen

**Affiliations:** ^1^Riddet Institute, Massey University, Palmerston North, New Zealand; ^2^Wageningen University and Research, Wageningen, Netherlands

**Keywords:** dietary protein, protein digestibility, DIAAS, PDCAAS, protein quality, plant-based protein, animal-based protein, protein metabolism

Amino acids found in food proteins are essential in the human diet not only for the maintenance of lean body mass and because of the involvement of amino acids in essential metabolic pathways, but also for modulating appetite and maintaining body weight, and optimal organ function, including muscle function. Optimal organ and muscle function underpin long term health.

Given projected world population growth, food protein demand, and the uncertainties in food production associated with global climate change and other drivers it is timely for an authoritative update on the subject of amino acids and protein in human nutrition.

It was in this context, and driven by the need for future world food and protein security coupled with environmental sustainability, that the international symposium “*Dietary Protein for Human Health*” organized by the Food and Agricultural Organization of the United Nations (FAO), the Riddet Institute, Massey University, Wageningen University and Research, and the International Atomic Energy Agency, was convened in Utrecht the Netherlands in September 2023. Themes covered at the Symposium included: protein nutrition and health; amino acid requirements; amino acid digestibility and availability; dietary protein quality including a review of the protein digestibility corrected amino acid score (PDCAAS) and digestible indispensable amino acid score (DIAAS) evaluation systems; the influence of protein quality on growth and development and on whole body protein metabolism; plant, animal and alternative proteins and their roles in sustainable nutrition; and future sustainable food protein production.

This Research Topic draws off the original research presented at the international symposium “*Dietary Protein for Human Health*” and the resultant collection of 25 scientific papers provides a comprehensive update of recent advances in the area.

The definition and quantification of protein and amino acid requirement values has long been contentious and uncertainty in this area still remains, with recent research pointing toward higher estimates of requirements. The ability of a food to deliver amino acids to meet a stated requirement has also been subject to intensive research over the years, though it has only been over the past decade that physiologically valid methods for determining amino acid digestibility and availability in humans have become generally available. The wider implications of amino acid uptake on growth and development in children and on body metabolism and organ and muscle function in adults remain important subjects of ongoing research. All of these topics are covered in depth. Recently the effect of climate change on food production, and at the same time the effect of food production systems on climate change itself have become hot topics for research, accompanied by societal calls for changes in consumption patterns of foods. Such recommendations certainly have implications for environmental sustainability, but also implications for nutritional sustainability, food affordability, and cultural mores. The challenge of adequately feeding the future world population is complex and multifactorial, and this complexity is addressed in the present Research Topic.

The Research Topic follows a progression of themes. Review papers by Calvez et al. and Wolfe et al. set the scene by establishing the overall relevance of a study of protein metabolism, protein nutrition and dietary protein quality. Other authors (Layman, Deutz et al., Trommelen and Loon, Groenendijk et al., Deane et al., Manary et al., Mensink) hone in on the specific roles of protein and amino acids in body protein turnover and muscle metabolism as well as malnutrition and disease states. Paoletti et al. and Moughan et al. provide an update on the estimation of amino acid requirements, while Gaudichon and Moughan and Lim address recent developments in protein quality scoring patterns and systems of evaluation. Two contributions (Hodgkinson, Kashyap et al.) address the *in vivo* determination of amino acid digestibility in humans, and the paper of Stein discusses the need for animal models of *in vivo* amino acid digestibility and reviews the evidence for choice of the growing pig as a valid model for the adult human. To allow for a more routine determination of amino acid digestibility in foods a validated *in vitro* digestibility assay is urgently needed. Three papers (Singh, Krul et al., Santos-Sánchez et al.) interrogate this topic. Stanton and Sheffield et al. focus on animal vs. plant foods as supplies of protein, amino acids, and other nutrients, while the works of Burlingame et al., Fletcher et al., Chungchunlam and Moughan offer an holistic assessment of the different dimensions of food sustainability. The Research Topic is completed with a paper (Xipsiti) providing an FAO perspective on protein quality evaluation and the establishment of an international database of food amino acid digestibility, looking to move the area forward and secure greater accuracy of amino acid provision.

Overall, the Research Topic adds to knowledge in a critical area. It remains important that we have a solid scientific evidence base to support amino acid requirement values that will reflect optimal metabolic function and health. Equally we need accurate information on how different foods and novel protein sources differ in their ability to provide the body with dietary essential amino acids. This has never been more important than now, with a significant global challenge to properly feed a growing human population within acceptable environmental boundaries.

